# Editorial: Advances in Immunotherapeutic Approaches to Tuberculosis

**DOI:** 10.3389/fimmu.2021.684200

**Published:** 2021-04-22

**Authors:** Iris Estrada García, Rogelio Hernández Pando, Juraj Ivanyi

**Affiliations:** ^1^ Department of Immunology, National School of Biological Sciences, ENCB-IPN, Mexico City, Mexico; ^2^ Experimental Pathology Section, Department of Pathology, National Institute of Medical Sciences and Nutrition ‘Salvador Zubiran’, Mexico City, Mexico; ^3^ Centre for Host–Microbiome Interactions, Guy’s Campus of Kings College, London, United Kingdom

**Keywords:** Tuberculosis, Intracellular infection, pulmonary infection disease, mycobacterial infections, Immunotherapies

Tuberculosis (TB) has been a widespread infectious disease since ancient times and still remains a major worldwide health problem. Significant progress in drug development during the last decade greatly contributed to TB therapy, while immunotherapies represent an interesting opportunity for further improving the control of TB. However, particular attention needs to be given to the increasing emergence of drug-resistant strains of *M. tuberculosis* (Mtb) and the association of TB with HIV infection and with chronic degenerative diseases such as type 2 diabetes. This special issue of the Frontiers contains a collection of 15 papers that advance various aspects that are relevant to TB immunotherapy. They can be grouped into three categories, concerning: i) role of innate immune cytokines, ii) recognition of specific antigenic determinants and iii) improvement of diagnostic assays. These papers are being briefly reported in the text and quoted in **Table 1**, listing the number of registered views so far for each.

**Table 1:** Review of Topic publications.

Category	Authors*	Subject	Views**
Role of innate immune cytokines	Cox–Keane	High IL-1b/low IL10 due to Histone deacetylase inhibitor	2,313
	Winchell–Flynn	IL-1 blockade, adjunct to chemotherapy	2,079
	Mishra–Khan	Protection by GM-CSF	1,515
	Yang–Dou	Protection by M1-cytokines	1,631
	Dutta–Schneider	Sex differences to therapies	784
Antigen-specific immunotherapy and prophylaxis	Deng–Yang	Mannosylated vaccine antigens	1,858
	Ortega–Espitia	Antibodies against T-cell epitopes	1,760
	Aguilo–Martin	Protective mucosal sIgA	1,597
	La Manna–Caccamo	Immunotherapy by genotype-unrestricted T cells	1,498
	Afkhami–Xing	Design of therapeutic vaccines	1,246
	Basile–Rottenberg	Protection by PD-1^+^ lung-resident T cells	1,110
	Camacho–Acosta	Sulfoglycolipid-CD1b specific antibodies	1,089
Diagnosis	Carranza–Torres	Distinguishing between active and latent TB	2,515
	Liu–Wang	New CirRNA biomarkers	1,409
	Chen–Fan	Low CD69+T cells post infection	1,517

*First and last authors; **Views registered on 2^nd^ April, 2021.

Much of the Mtb infection cycle is fulfilled in the cytoplasm of macrophages, which represent the key innate immune cellular element involved in the infection. A study of glycolysis and proinflammatory cytokines is reported on Mtb infected human lung macrophage populations *in vitro* (Cox et al.). Inhibition of histone deacetylase by suberanilohydroxamic acid increased interleukin (IL-1b) and reduced IL-10 production, thus enhancing interferon-γ, (IFN-γ), tumour necrosing factor-α (TNF−α) and granulocyte-macrophage colony-stimulating factor (GM-CSF) co-production in responding T helper cells. These results indicate that modulation of innate immunometabolic processes in macrophages can promote subsequent adaptive proinflammatory responses. A study of Linezolid chemotherapy in mice and macaques investigated reducing its IL-1 mediated bone marrow toxicity and TB pathology. Lung inflammation could be reduced using an IL-1 receptor antagonist Anakinra (Winchell et al.), without being immunosuppressive and retaining its therapeutic impact on TB pathology The role of GM-CSF for innate resistance against TB was suggested on the grounds of its higher production by Mtb-infected human macrophages (Mishra et al.). The increased expression of genes involved in the GM-CSF signalling pathway was associated with diminished Mtb growth, hence indicating its translational potential as a host directed therapy. A subset of lung macrophages, linked to higher Th1 and Th17 responses, has been associated with better protection in Bacillus Calmette Guerin (BCG) vaccinated mice (Yang et al.). The role of sex influences on adjunctive anti-inflammatory therapies of TB has been examined, using monocyte derived macrophages (Dutta and Schneider).

Bacterial or immune cell derivatives could represent another form of immunotherapy. The immunogenicity of 15 secreted O-mannosylated glycoproteins of BCG was examined following their purification and expression in *Mycobacterium smegmatis* and *Escherichia coli* recombinants (Deng et al.). Two glycoproteins, BCG_0470 and BCG_0980 (PstS3), were proposed for vaccine development on the grounds of inducing higher immunodominant Th1 responses. A novel approach is represented by the demonstration of single domain antibody ligands with T-cell receptor (TcR)-like specificity (Ortega et al.). A clone, selected from a phage display library, recognised an HLA-A*0201 bound peptide, which is known to be an immunodominant epitope of the Ag85B antigen for human T cells. This proof-of-principle for generating antibodies with TcR-like specificity against epitopes on the surface of both replicating and dormant Mtb infected macrophages, warrants further investigation, since such antibodies, particularly when conjugated with apoptosis-inducing ligands, could lead to passive immunotherapy in immunocompromised HIV-infected TB patients. Further synergistic benefit may come from agents, e.g. IFNg, which enhance the surface expression of major histocompatibility complex (MHC) -bound epitope targets. Vaccination, through respiratory delivery of a whole cell inactivated vaccine, showed improved polymeric Ig receptor (i.e. pIgA dependent) protection in mice and non-human primates (Aguilo et al.). The immunotherapeutic potential of ‘unconventional T cells’ has been considered for cell transfer therapy of severe multidrug resistant infection (La Manna et al.). This included CD1–restricted T cells, MHC-related protein-1–restricted mucosal-associated invariant T cells, MHC class Ib–reactive T cells, and γδ T cells. A review of therapeutic vaccines covered several aspects of their construction. Potentials for faster disease resolution (Afkhami et al.) and shortening of chemotherapy for drug-resistant strains, fragmented/incomplete therapy and against relapsing disease, have been evaluated. The mechanisms discussed pertain to distinct immune responses induced by the infection versus the vaccines, as well as their efficacy in HIV co-infected individuals. The importance of non-recirculating, lung-residing immunity was suggested by the finding that IFN-γ secreting PD-1+ T cells accumulate in the lungs following aerosol and intratracheal delivery, but not in the lymph nodes and not after subcutaneous inoculation of BCG (Basile et al.). Single chain T cell receptor and light chain domain antibody ligands produced by phage display technology recognized the Mtb-specific sulfoglycolipid Ac_2_SGL, complexed with the monomorphic CD1b cellular receptor, which are recognized by T cells in both active and latent TB (Camacho et al.). Molecular modelling suggested better recognition of the natural complex than it’s synthetic analogue and this difference was interpreted as support for the therapeutic and diagnostic targeting of Mtb infected cells.

The prospects for IGRA and QTF-Plus tests distinguishing between active and latent TB has been reviewed with the aim of improving the specificity of tuberculin skin testing, which is obscured by BCG vaccination (Carranza et al.). Moreover, the frequency of the proportion of TNF-a only T_EFF_ cells, with an effector memory phenotype (CD45RA^−^CCR7^−^CD127^−^) was associated with a higher risk of progression to active TB. However, expansion of immature myeloid-derived suppressor cells has been linked to both active TB and recently acquired latent TB. Differential diagnosis involved both cellular and antibody responses to the DosR regulon encoded latency-associated antigens and IFN-γ signalling, and miRNA and molecular signatures of the blood transcriptome. A study of circRNA microarray expression profiles identified circRNA_051239, circRNA_029965, and circRNA_404022 as potential biomarkers for TB diagnosis (Liu et al.). The expression of the CD69 biomarker of T-cell activation in active and latent TB cohorts was shown to distinguish latently infected subjects from those who were uninfected, despite continuous exposure to infection (Chen et al.).

In conclusion, the present collection provides further evidence that the immune system is a significant factor in both the containment and cure of Mtb infection. Immunotherapy based on the augmentation of protective immunity or decreasing the immune modulatory responses during late progressive disease can be of value as an adjunctive agent in TB treatment. In this respect, novel combinations of both new and old drugs need to be evaluated for their efficacy, when combined with various forms of immunotherapy. The goals of future immunotherapies must be to shorten chemotherapy use, prevention latent TB reactivation and prevent transmission of the infection.

## Author Contributions

IEG, RHP, and JI wrote and reviewed the manuscript. All authors contributed to the article and approved the submitted version.

## Conflict of Interest

The authors declare that the research was conducted in the absence of any commercial or financial relationships that could be construed as a potential conflict of interest.

